# Iron(III)-Tannic Molecular Nanoparticles Enhance Autophagy effect and T_1_ MRI Contrast in Liver Cell Lines

**DOI:** 10.1038/s41598-018-25108-1

**Published:** 2018-04-27

**Authors:** Krungchanuchat Saowalak, Thongtem Titipun, Thongtem Somchai, Pilapong Chalermchai

**Affiliations:** 1 Center of Excellence for Molecular Imaging (CEMI), Department of Radiologic Technology, Faculty of Associated Medical Sciences, Chiang Mai University, Chiang Mai, 50200 Thailand; 2 Department of Chemistry, Faculty of Science, Chiang Mai University, Chiang Mai, 50200 Thailand; 3 Department of Physics and Materials Science, Faculty of Science, Chiang Mai University, Chiang Mai, 50200 Thailand

## Abstract

Herein, a new molecular nanoparticle based on iron(III)-tannic complexes (Fe–TA NPs) is presented. The Fe–TA NPs were simply obtained by mixing the precursors in a buffered solution at room temperature, and they exhibited good physicochemical properties with capability of inducing autophagy in both hepatocellular carcinoma cells (HepG2.2.15) and normal rat hepatocytes (AML12). The Fe–TA NPs were found to induce HepG2.2.15 cell death via autophagic cell death but have no effect on cell viability in AML12 cells. This is possibly due to the much higher uptake of the Fe–TA NPs by the HepG2.2.15 cells via the receptor-mediated endocytosis pathway. As a consequence, enhancement of the T_1_ MRI contrast was clearly observed in the HepG2.2.15 cells. The results demonstrate that the Fe–TA NPs could provide a new strategy combining diagnostic and therapeutic functions for hepatocellular carcinoma. Additionally, because of their autophagy-inducing properties, they can be applied as autophagy enhancers for prevention and treatment of other diseases.

## Introduction

Autophagy is a crucial biological process of the cellular clearance pathway of degradation of damaged biomolecules or organelles and then recycling of these damaged biomolecules or organelles as biological resources for other essential biological pathways^1–3^. A number of evidences support the role of autophagy in sustaining cell survival as well as inducing cell death^4^. Lack of autophagy is associated with increased risk of diverse diseases^5,6^. At the same time, excess autophagy and/or deregulated autophagy can result in cell death, the so-called autophagy cell death (ACD)^7,8^. Consequently, targeting autophagy is an emerging strategy for drug discovery. Various works have demonstrated that modulation of autophagy plays a crucial role in the neuroprotective effects of Alzheimer’s and Parkinson’s diseases, and in cancer chemoprevention and treatment^9–11^. Thus, autophagy modulation is a promising approach to control the cellular biology associated with the prevention and treatment of a diverse variety of diseases.

It is well-known that nutrient starvation is one of the most common routes to induce autophagy. Unfortunately, the major concern about using starvation for medical purposes is its relevance to clinical practice^12^. Apart from starvation, external stimulation by autophagic modulators has also been reported. The authors came across related studies, and it was found that a number of autophagic modulators, ranging from synthetic molecules to natural products, have been developed^13–15^. Several nanoparticles have also been found to induce the autophagy process. Nanoparticle-based autophagic modulators are of great interest because they not only exhibit autophagy-inducing property but also can be multi-functionalized with imaging moieties^16–19^. Therefore, they are considered additionally beneficial for real-time monitoring of their action *in vivo*.

Recently, molecular nanoparticles assembled from small molecules through non-covalent interactions have attracted considerable attention in various fields^20–24^. Among them, molecular nanoparticles of metal-polyphenol are particularly of interest in both disease diagnosis and treatment because they can integrate imaging moieties and therapeutic agents within the same platform. It is well-known that various metals are utilized for imaging in various techniques: for example, Cu and Ga for positron emission tomography (PET) scan; Gd, Mn, and Fe for magnetic resonance imaging (MRI); and Re, Eu, and Tb for optical imaging^19,25,26^. Among them, paramagnetic Fe is of great interest because of its imaging capability in MRI with less toxicity. Tannic acid (TA) is a typical kind of large natural polyphenol. It is considered as a polydentate ligand that can bind to various metal ions, especially ferric ion, to form high stable metal complexes^27^. Additionally, unlike a small polyphenol, interaction of ferric ions and tannic acid has shown unique properties such as iron-mediated self-assembly of Fe–TA complexes, resulting in molecular nanoparticles of Fe–TA complexes^28^. The large Fe–TA complexes are critical for enhancing the MRI signal because large paramagnetic molecular nanoparticles have good capability of enhancing the rate of water–proton exchange by slowing down the rotational diffusion^29^. From a therapeutic point of view, TA has been shown to have a variety of beneficial effects on health, which are primarily related to its antioxidant and anticancer properties. TA was found to exhibit anti-proliferative activity in several cancer cell lines, induce cancer cell apoptosis and autophagic cell death^30–34^. The abovementioned rationales substantiate the integration of the imaging capability (in MRI) of the paramagnetic ferric ions and the therapeutic potential of tannic acid in the form of its molecular nanoparticles, which allows for its use in imaging, prevention and treatment.

In this study, nanoparticles of Fe–TA complexes with good physicochemical properties were developed using a low-cost, reproducible method. Whether the Fe–TA NPs induced cellular autophagy process was investigated. Two different liver cell lines, hepatocellular carcinoma cells (HepG2.2.15) and normal rat hepatocyte (AML12), were selected as *in vitro* models to comparatively study the autophagy effect based on their interactions and biological responses to the Fe–TA NPs. Apart from the therapeutic potential of Fe–TA NPs with regard to biological effects, the possible use of Fe–TA NPs for enhancement of the MRI signal was also investigated. The findings of this study might give a new insight into treatment and imaging of liver cancer, and the Fe–TA NPs would hopefully be applicable as autophagic modulators in other cells.

## Results and Discussion

### Large scale, reproducible preparation of Fe–TA NPs can be easily achieved, and they exhibit good physicochemical properties

**Figure 1:**
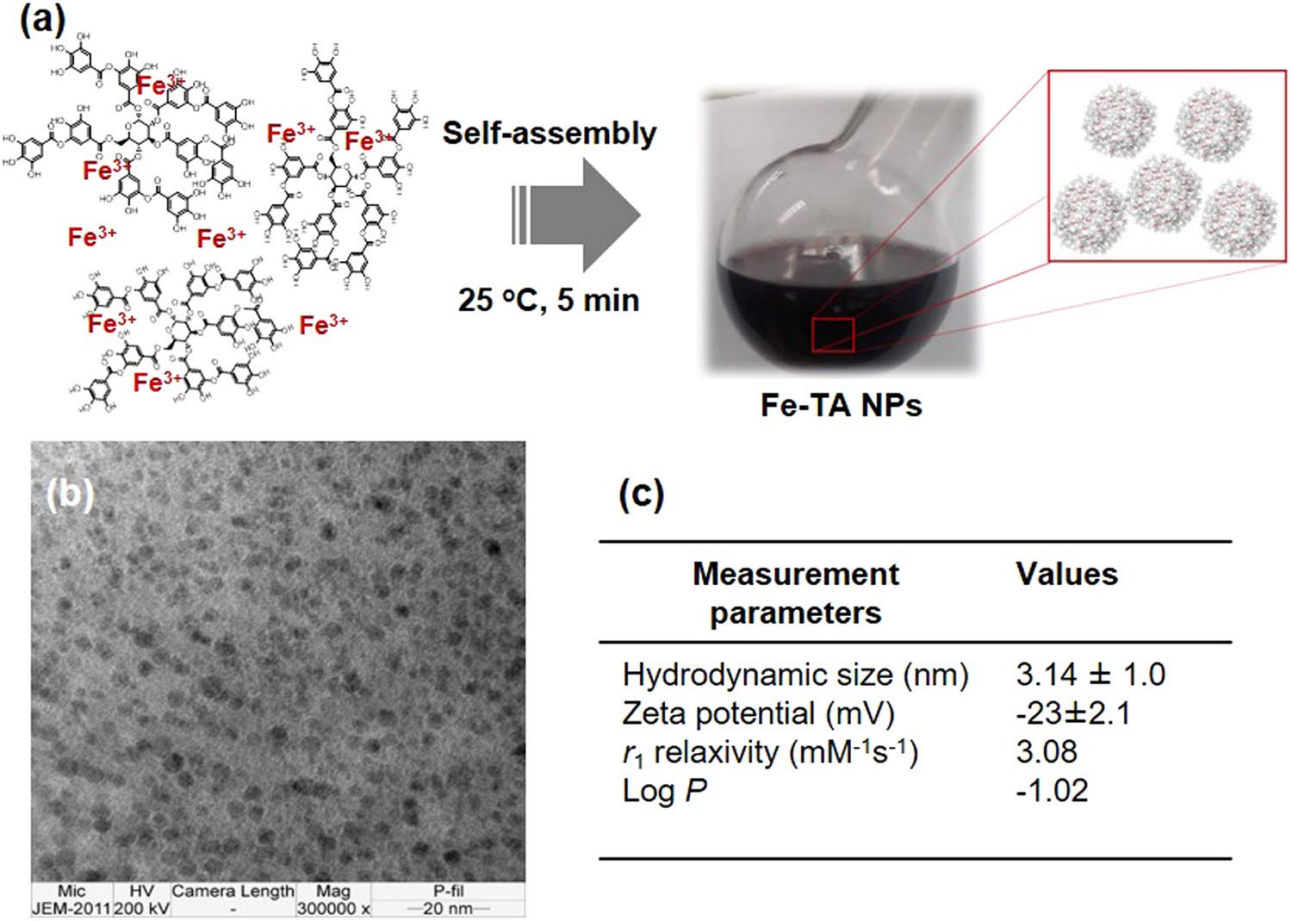
(**a**) Schematic illustration of the preparation of Fe–TA NPs, (**b**) the TEM image of Fe–TA NPs, (**c**) typical characteristics of Fe–TA NPs.

The Fe–TA NPs were easily obtained by mixing ferric chloride and tannic acid in PBS buffer (pH 7.4) at room temperature for a few minutes in ambient air. Schematic illustration of the preparation of Fe–TA NPs is shown in Fig. 1a. Under this condition, Fe–TA complexes undergo an iron-mediated self-assembly process to form nanosized Fe–TA complexes. It should be noted that PBS buffer (pH 7.4) was chosen as the reaction medium because this condition was suitable to form predominantly Tris-coordinated Fe–TA NPs (having a more stable structure)^28,35^.

The characteristics of the Fe–TA complexes were confirmed by observing the UV-Vis charge transfer (CT) band at ~525 nm (Figure S1a) and the vibrational peaks of TA, as well as the Fe-O bonding (Figure S1b–d)^36,37^. In addition, the XPS analysis also confirmed the characteristic types of bonding found in Fe–TA NPs (Figure S2)^38,39^. The TEM images (Fig. 1b) reveal that Fe–TA NPs have a spherical shape with diameters in the range of ~2–5 nm. Other physicochemical properties and longitudinal MRI relaxivity were investigated, and the findings are summarized in Fig. 1c. The hydrodynamic diameter (HD) and the zeta potential (ZP) were determined as 3.14 ± 1.0 nm and −23 ± 2.1 mV, respectively. The large negative zeta potential indicates good colloidal stability of the Fe–TA NPs in the aqueous medium^40^. This result is in consistent with the measured log P value of −1.0249, indicating good water solubility^41^. Previously, it has been demonstrated that molecular nanoparticles of Fe–TA complexes exhibit paramagnetism and enhance MRI signal intensity in T_1_-weighted imaging^28^. Similarly, the obtained Fe–TA NPs were also found to induce signal enhancement in T_1_-weighted images, with *r*_1_ values of 3.08 mM^−1^ s^−1^ (in 4% acrylamide gel phantom), indicating that it can be used for increasing the sensitivity of MRI.

**Figure 2:**
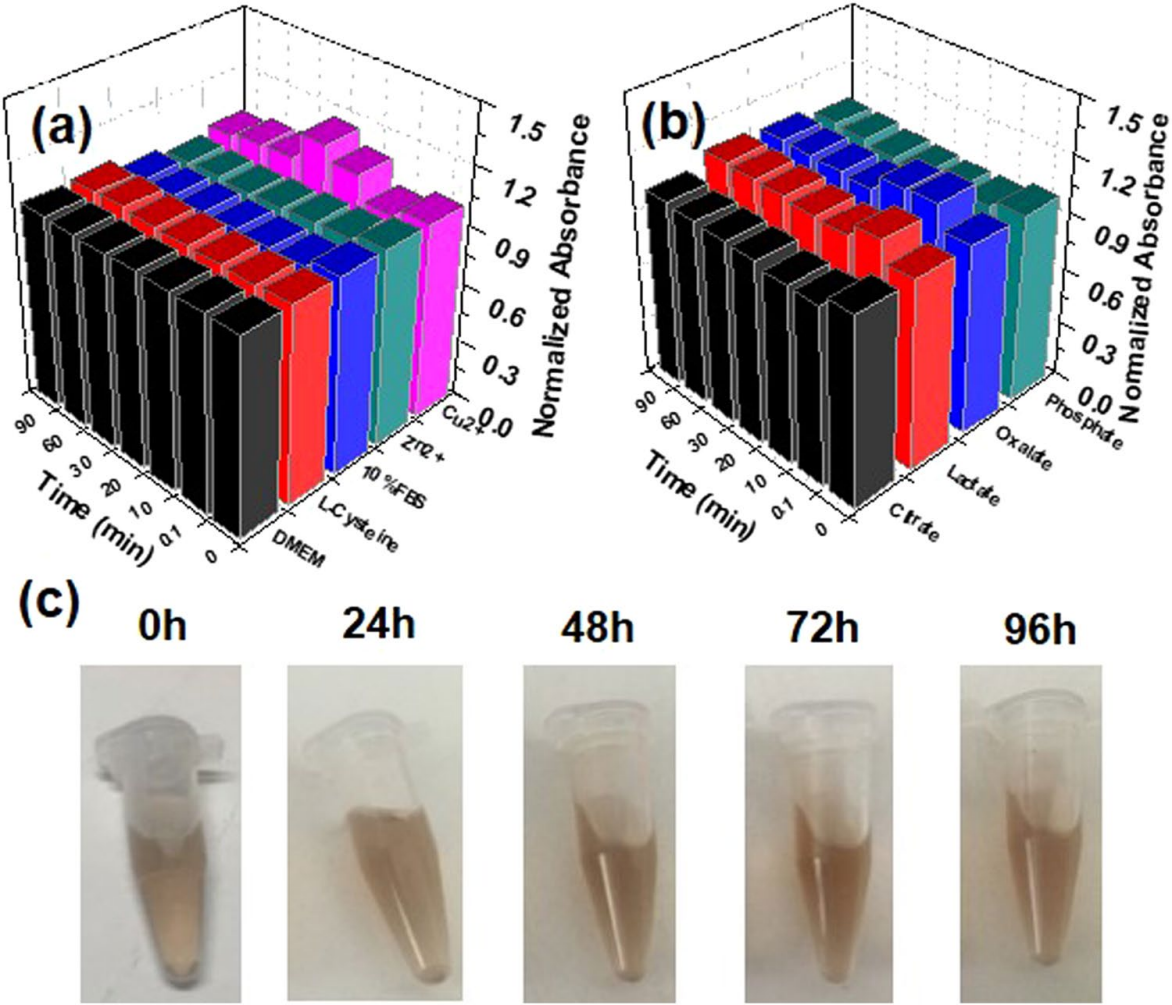
(**a**,**b**) Normalized absorbance of Fe–TA NPs in various competitive solutions. (**c**) Suspension stability of Fe–TA NPs in 100% FBS at different lengths of time.

As far as stability is concerned, transchelation and transmetallation of the Fe–TA NPs by endogenous ligands and metals were taken into account. Generally, several oxoanionic ligands (e.g., citrate, oxalate, phosphate, and lactate anions), biomolecules (in culture medium and serum), and endogenous metal ions (e.g., Cu^2+^, Zn^2+^) are found to exhibit competitive binding to the ferric ions or the tannic acid in the Fe–TA NPs. Therefore, in this study, the Fe–TA NPs were challenged with solutions containing competitive molecules, for example, L-cysteine, Cu^2+^, Zn^2+^, various oxyanions, DMEM solution, and 10% FBS solution, over a period of time. The results from the stability tests (Fig. 2a,b) show that the Fe–TA NPs were highly stable against transchelation and transmetallation. The high stability of Fe–TA NPs is associated with their high K_d_ complex as well as their dominant structure being a Tris-coordinated structure, which is more stable complex. To further explore the stability of Fe–TA NPs, they were incubated with 100% FBS at 37 °C for different lengths of incubation time. It was found that the Fe–TA NPs remained stable over 96 h without any aggregation (Fig. 2c). Furthermore, time-dependent HD of the Fe–TA NPs in PBS containing 10% FBS was also analyzed using DLS to observe the change of HD as well as their stability in physiological buffer. The results (see Figure S3) show no significant change in HD of the Fe–TA NPs after incubation in PBS containing 10% FBS over a period of time, indicating good colloidal stability in physiological buffer.

### Fe–TA NPs are capable of inducing autophagy in liver cell lines

**Figure 3:**
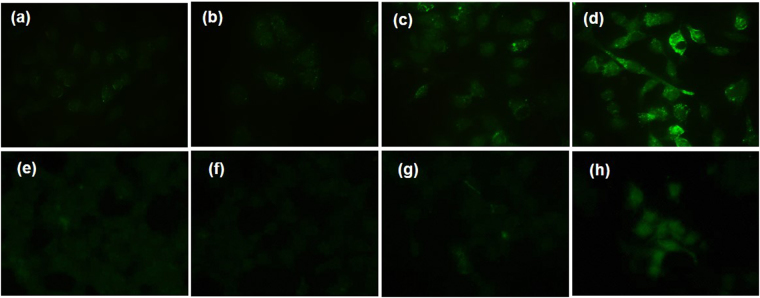
Fluorescence microscope imaging of immunofluorescence staining of LC3B of HepG2.2.15 cells (**a**–**d**) and AML12 cells (**e**–**h**) after 24 h of treatment; (**a,e**) untreated cells, (**b**,**f**) pure TA treated cells (30 µM), (**c,g**) 10 µM Fe–TA NPs treated cells (3 µM in TA equivalent), and (**d,h**) 100 µM Fe–TA NP treated cells (30 µM in TA equivalent).

**Figure 4:**
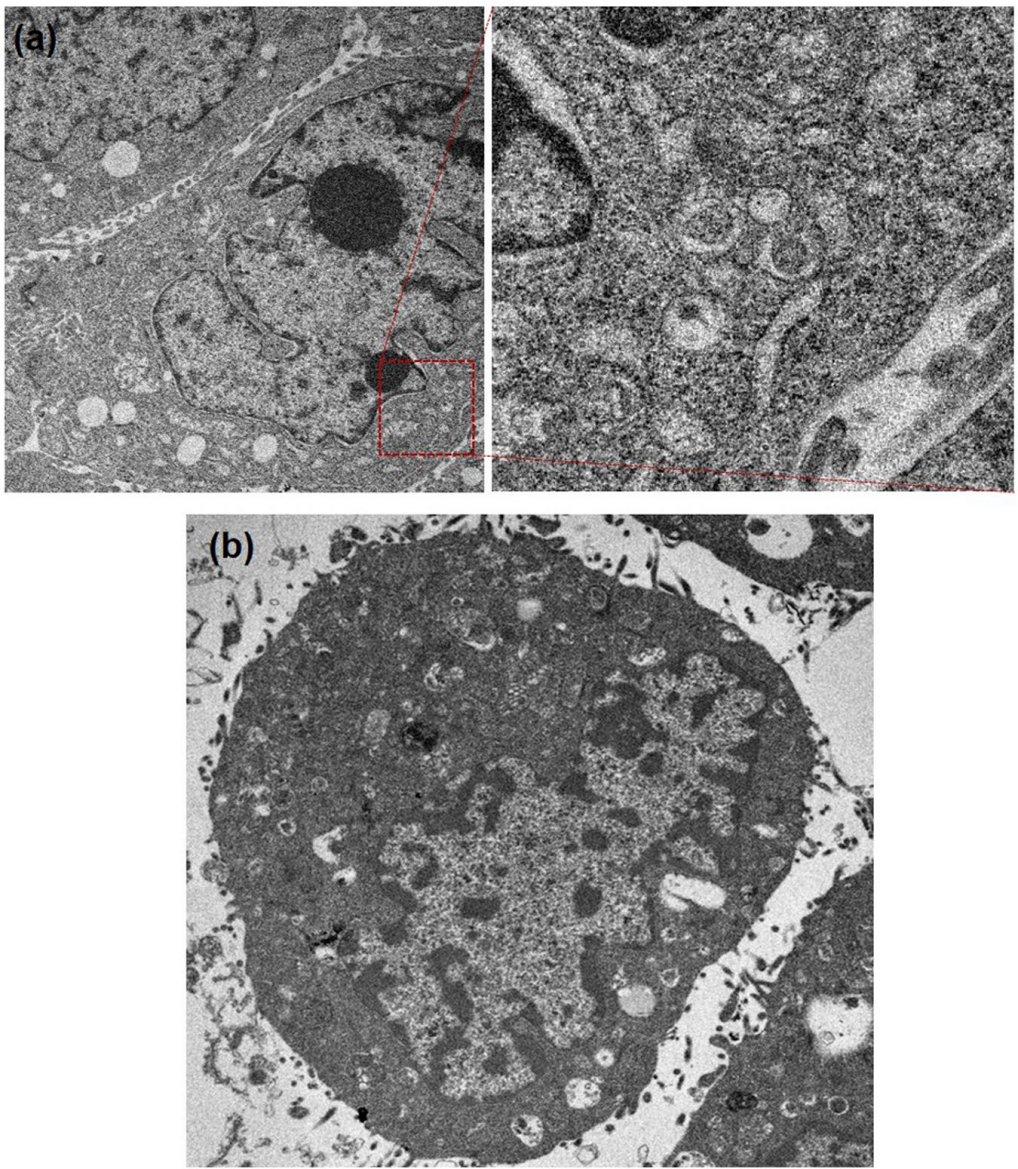
TEM analysis of autophagosomes of (**a**) HepG2.2.15 cells and (**b**) AML12 cells after being treated with 100 µM Fe-TA NPs for 24 h.

To test whether Fe–TA NPs are capable of inducing autophagic effect in liver cell lines, immunofluorescence staining of microtubule-associated protein light chain 3 (LC3) and TEM analysis were initially carried out. LC3 is known as a specific marker for the early stage of autophagy. Generally, during autophagy, endogenous LC3 can be visualized either as diffuse cytoplasmic pool or as punctate structures^42^. As shown in Fig. 3, green fluorescence signals of LC3B are apparently observed in the treated cells (both HepG2.2.15 cells and AML12 cells), compared to those observed in pure TA treated cells and untreated cells. Notably, it is more clearly observed in HepG2.2.15 cells in a concentration-dependent manner. From this result, it can be inferred that Fe–TA NPs are capable of inducing the autophagy process in both cancer and normal cell lines with different degrees of induction. TEM analysis was carried out in order to observe autophagosomes (double-membrane vesicle) within the cells. As seen in the TEM images (Fig. 4), the autophagosomes were observed in both the cell lines as well. In addition, the levels of LC3 mRNA in HepG2.2.15 cells were also investigated and the results are shown in Figure S4. The relative LC3 mRNA expressions of the Fe–TA NPs treated were significantly higher than that in the control (especially in the 100 µM treated), confirming the induction of autophagy by the Fe–TA NPs.

### Fe–TA NPs were found to induce liver cancer cells death (HepG2.2.15) but not in normal hepatocyte cells (AML12)

**Figure 5:**
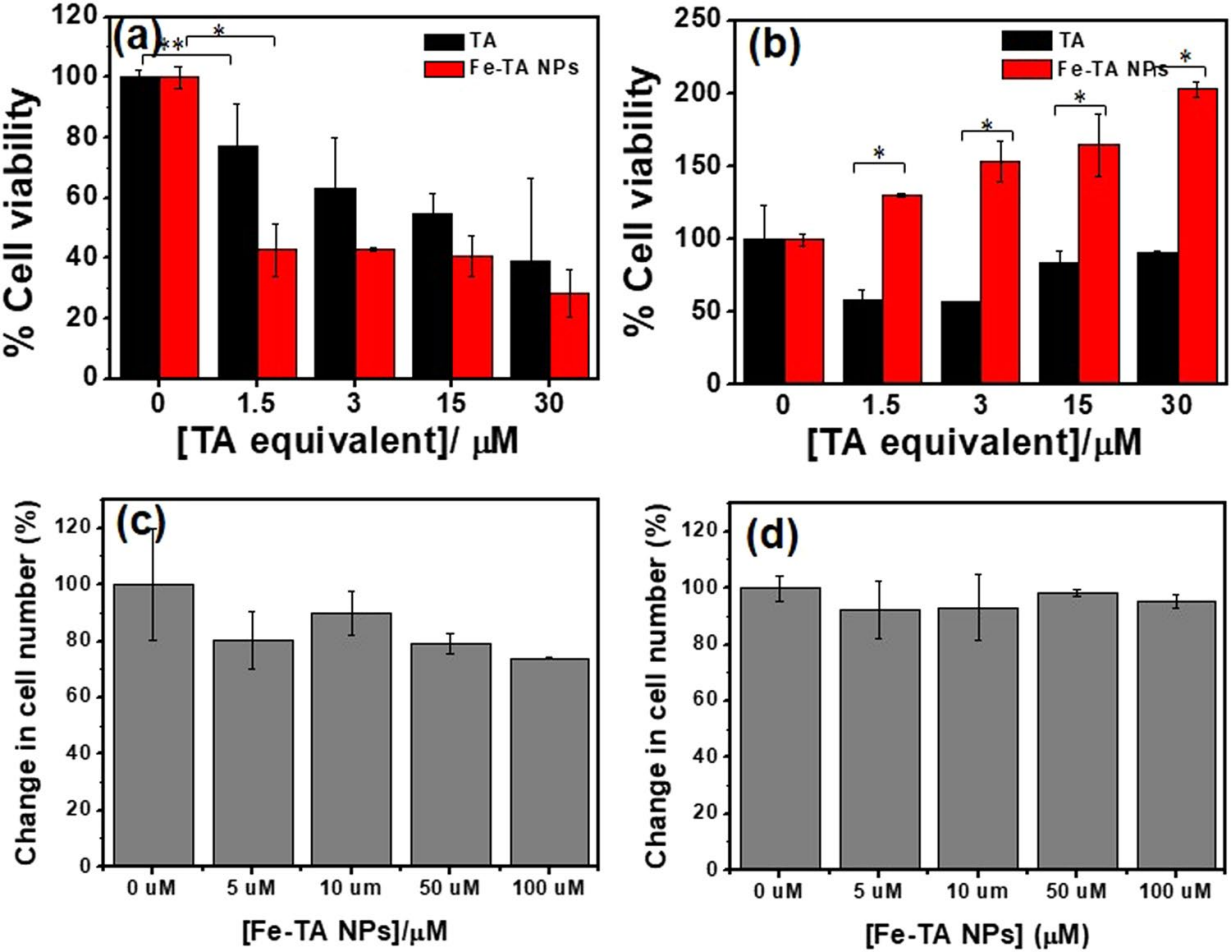
MTT assay of (**a**) HepG2.2.15 cells and (**b**) AML12 cells after treatment with pure TA and Fe–TA NPs for 24 h. Cell counting assay of (**a**) HepG2.2.15 cells and (**b**) AML12 cells after treatment with pure TA and the Fe–TA NPs for 24 h. (*p < 0.05, **p > 0.05).

**Figure 6:**
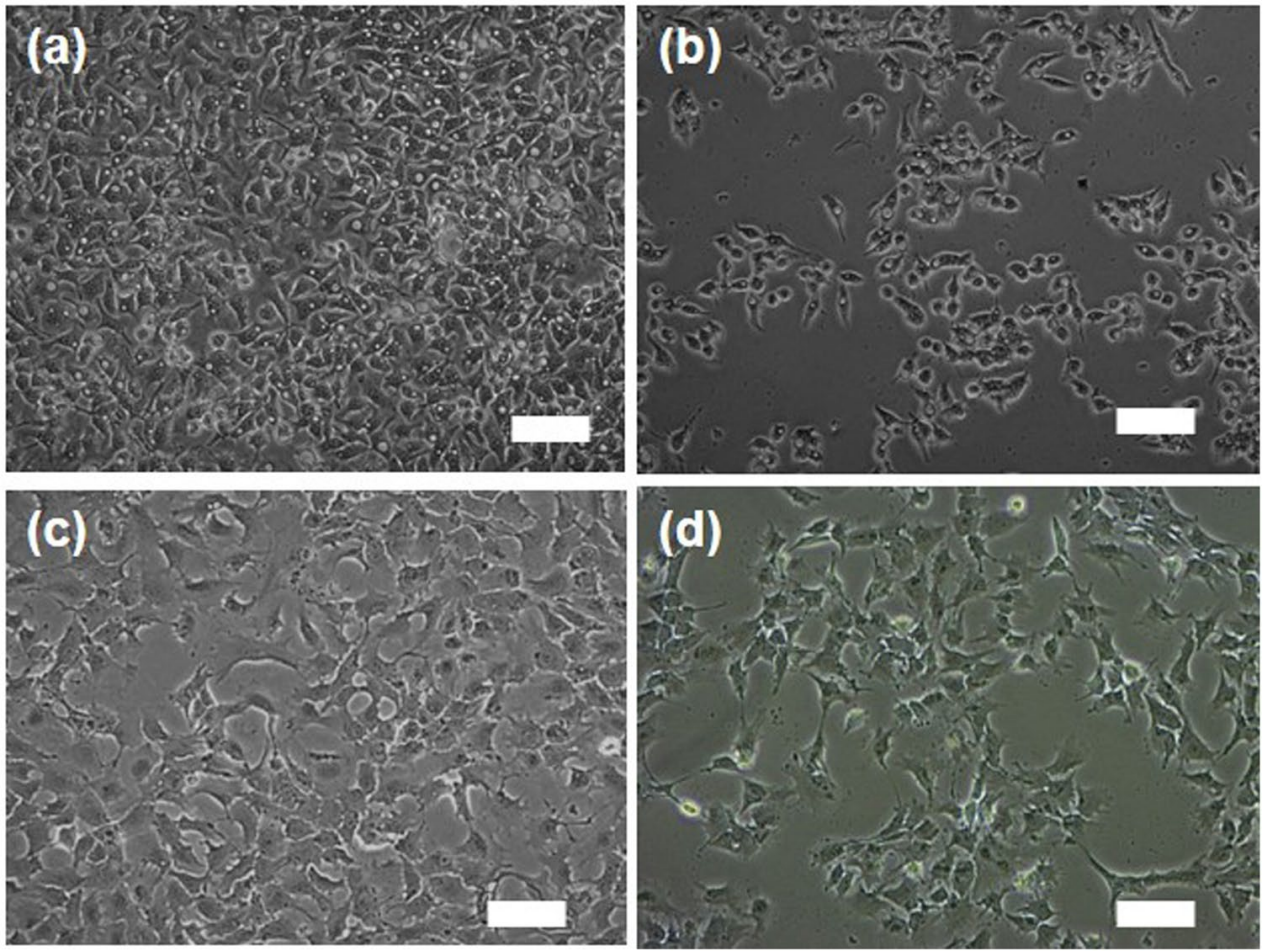
Microscope images (phase contrast) of (**a**) untreated HepG2.2.15 cells, (**b**) 100 µM Fe–TA NPs treated HepG2.2.15 cells, (**c**) untreated AML12 cell and (**d**) 100 µM Fe–TA NPs treated AML12 cell for 24 h (Scale bar = 50 µM).

Next, a comparative study of the Fe–TA NP mediated cytotoxicity and biophysical change between HepG2.2.15 cells and AML12 cells was conducted. First of all, MTT assay was carried out, and the results are shown in Fig. 5a,b. In HepG2.2.15 cells, surprisingly, Fe–TA NPs were found to exhibit better anti-proliferative activity than pure TA. In the case of AML12 cells, the Fe–TA NPs did not exhibit cytotoxicity but, instead induced cell proliferation. However, TA was found to induce cytotoxicity. These contradictory results of Fe–TA NPs on cellular cytotoxicity observed in different liver cell lines may have beneficial therapeutic effects for cancer selective treatment. Next, the cell counting assay was conducted to assess the cytotoxicity and to also observe the biophysical change (cell morphology) after treatment. As expected, the number of HepG2.2.15 cells decreased as the concentration of the Fe–TA NPs increased, but the number of AML12 cells remained unchanged at all tested concentrations (Fig. 5c,d). However, it should be noted that the cell counting assay adopted in this study was not a highly sensitive assay to assess cytotoxicity, as compared to the MTT assay, but it was suitable for investigating the biological effects including the biophysical and the biochemical changes before cell death. As is known, cell morphology is a crucial biophysical property in the study of cellular interaction and biological response. Particular changes in the molecular levels of cells lead to change in cell morphology^43^. Herein, the effects of Fe–TA NPs on the morphological changes of the two cell lines were assessed. As seen in Fig. 6 and Figure S5, change in cell morphology could be clearly observed in the treated HepG2.2.15 cells, but there was no significant change observed in the treated AML12 cells. This indicates that biological responses to the Fe–TA NPs in the two different cells are totally different. The next attempt was to find out why Fe–TA NPs affect the cells differently.

### Biological response to Fe–TA NPs is dependence on cellular uptake capability

**Figure 7:**
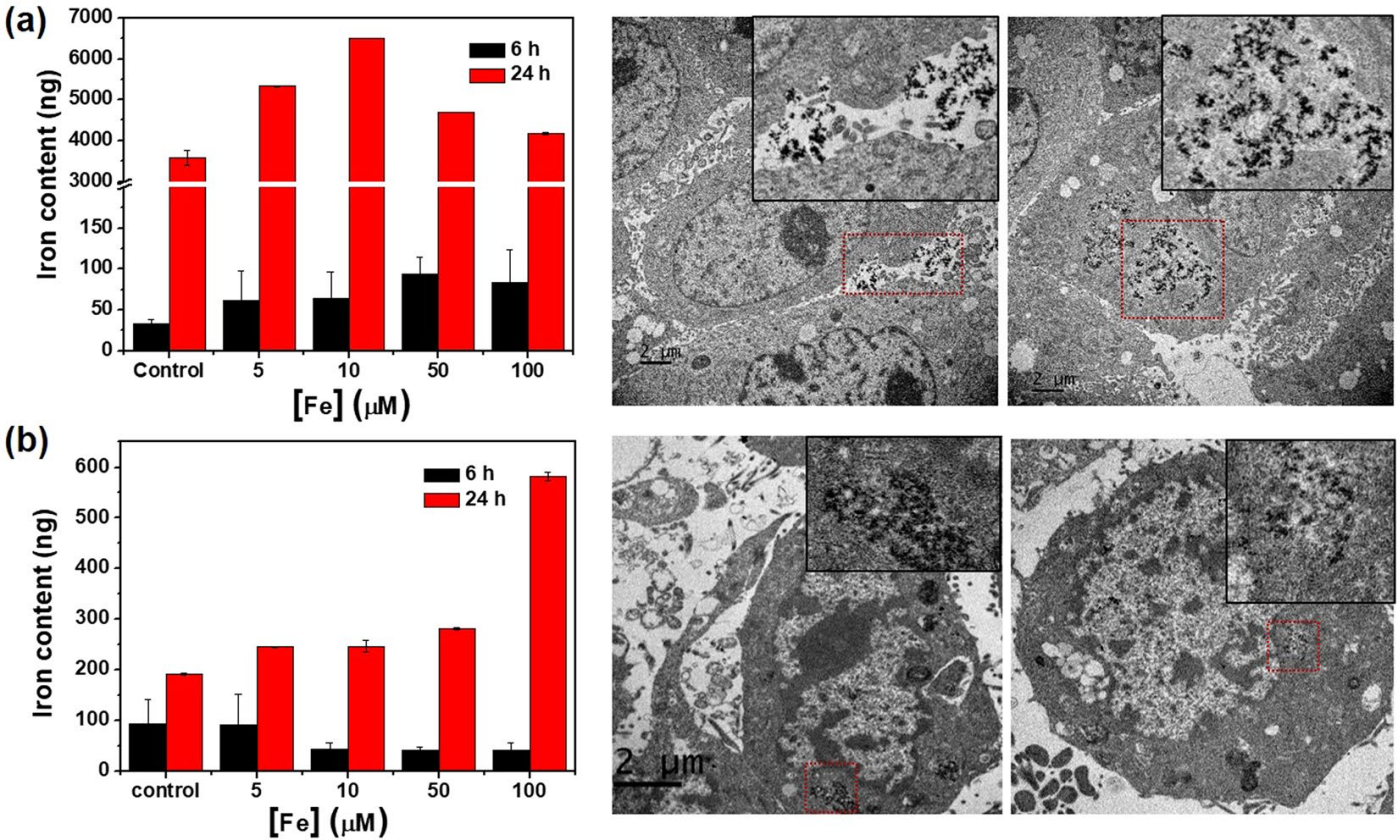
Intracellular iron content and TEM analysis of intracellular Fe–TA NPs (24 h of treatment) of (**a**) HepG2.2.15 cells and (**b**) AML12 cells.

**Figure 8:**
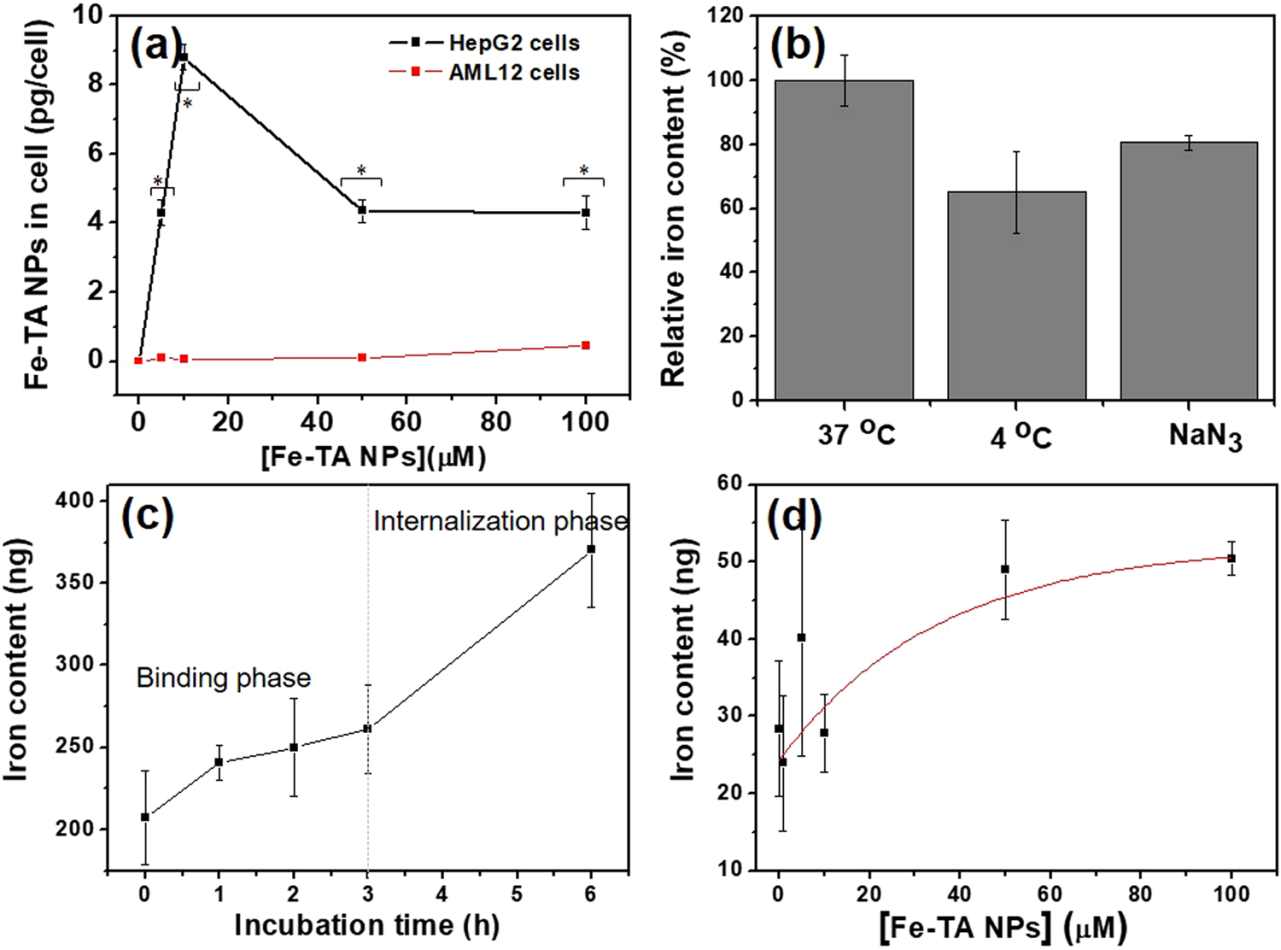
(**a**) The amount of Fe–TA NPs presented within the cells after treatment with different concentrations of Fe–TA NPs for 24 h. (**b**) The relative intracellular iron content of HepG2.2.15 cells treated with Fe–TA NPs at 37 °C with/without endocytosis inhibitor, and at 4 °C. (**c**,**d**) The Fe–TA NPs uptake in HepG2.2.15 cells after being treated in a time and concentration-dependent manner. (*p < 0.05).

Next, the intracellular accumulation of Fe–TA NPs in the cells was investigated by measuring the total iron content. Apparently, Fe–TA NPs can be taken up by HepG2.2.15 cells in concentration and in a time-dependent manner, as compared to untreated HepG2.2.15 cells (Fig. 7a). It should be noted that the iron content was found to decrease after incubating with higher concentrations of the Fe–TA NPs (>10 μM) at 24 h. This is possibly due to fewer numbers of viable cells being measured (Fe–TA NP induced cell death) and/or exocytosis of Fe–TA NPs. As for AML12 cells, enhancement of the intracellular iron content was not observed after incubation with different concentrations of Fe–TA NPs at 6 h, but the intracellular iron content was found to increase in a concentration-dependent manner at 24 h (Fig. 7b), indicating that the Fe–TA NPs were also taken up by the normal hepatocyte. The corresponding TEM images of the treated cells (both HepG2.2.15 and AML12 cells) clearly demonstrate that the Fe–TA NPs were internalized into the cells and distributed mainly in the cytoplasm. Next, a comparative analysis of the exact amount of Fe–TA NPs within the cells was attempted in order to determine the uptake efficiency of the two different cell lines. As shown in Fig. 8a, HepG2.2.15 cells had more efficiency regarding cellular uptake of Fe–TA NPs than AML12 cells. From the results mentioned above, it can be inferred that toxicity-related biological responses to Fe–TA NPs are closely related to the uptake efficiency of the particular cell.

Because the Fe–TA NPs were capable of inducing cytotoxicity in the HepG2.2.15 cells via their enhanced cellular uptake, an attempt was made to figure out the uptake mechanism in HepG2.2.15 cells. According to the TEM imaging data, cellular membrane invagination containing a number of the Fe–TA NPs could be observed in the treated HepG2.2.15 cells (Fig. 7a). Possibly, cellular uptake may be mediated by the endocytosis pathway. In order to determine whether internalization of the Fe–TA NPs was mediated by such a pathway or not, cellular uptake experiments were conducted under the conditions of cellular ATP depletion. As expected, pretreatment with the ATP-depleted agent (NaN_3_) and incubation at 4 °C reduced the intracellular iron content, compared to the baseline level (incubated at 37 °C), thus confirming the uptake mechanism of endocytosis (Fig. 8b). There have been reports that cell surface receptors play a role in facilitating the cellular uptake kinetic in various nanoparticles^44,45^. An attempt was made in this study to determine whether the uptake of the Fe–TA NPs into the HepG2.2.15 cells was associated with receptor-mediated uptake or not. Cellular uptake kinetic was performed by incubating the HepG2.2.15 cells with the Fe–TA NPs (10 μM) for 0–6 h (with 100% cell viability). The amount of Fe–TA NPs in the HepG2.2.15 cells treated for different lengths of time shows two distinct phases (see Fig. 8c). The initial phase of the uptake curve (first 3 h) represents the Fe–TA NPs on the cell membrane. The later phase occurring after 3 h indicates the internalization of the Fe–TA NPs. Additionally, iron content measurement was also conducted in a concentration-dependent manner during the binding event (3 h). The result shows the typical Langmuir binding kinetic for ligand–receptor interactions, at which the saturated pattern indicates the “receptor-limited” regime in the presence of excess Fe–TA NPs (Fig. 8d). However, it is still unclear what the receptor gets involved. More research is necessary before considering the utilization of Fe–TA NPs for targeted imaging and treatment of HCC.

**Figure 9:**
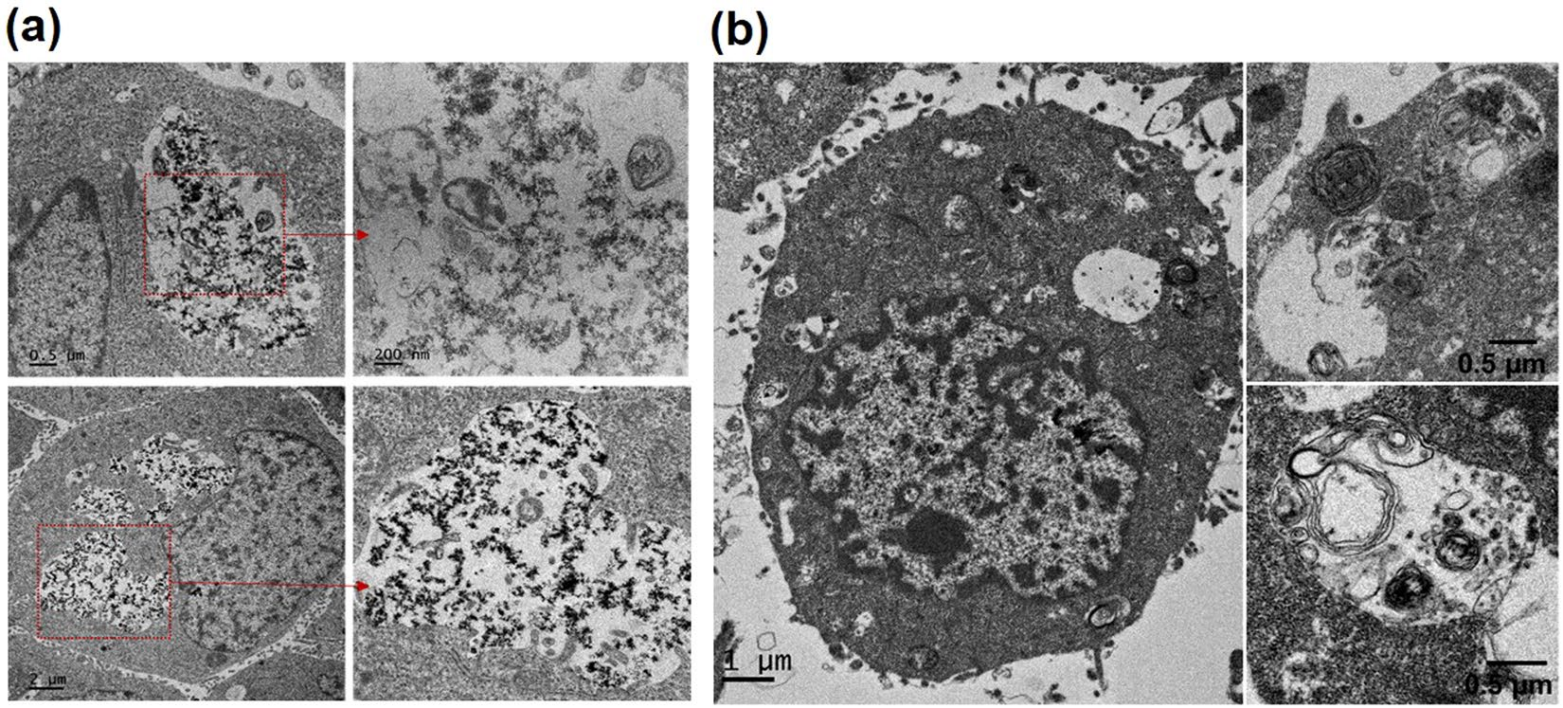
TEM analysis of vesicular structures in (**a**) 100 µM Fe–TA NPs treated HepG2.2.15 cells and (**b**) 100 µM Fe–TA NPs treated AML12 cells for 24 h.

From the results mentioned above, it is evident that cytotoxicity is closely related to cellular uptake capability. We further explored why the internalized Fe–TA NPs induced HepG2.2.15 cell death. According to the results of the TEM analysis (Fig. 9a), large vesicular structures containing Fe–TA NPs and biologic materials were clearly observed. In addition, abnormality in the activity of acidic organelles (e.g., lysosomes) was clearly observed upon using the cytotoxic dose of Fe–TA NPs (100 µM) for treating HepG2.2.15 cells (Figure S6), but no effects were found on cell cycle disruption and ROS over-generation. Instead there was reduced intracellular ROS (see Figure S7a–c). The enhanced activity in the acidic organelles indicates excessive self-digestion, which results in autophagic cell death (ACD). This might be a key mechanism in Fe–TA NP induced HepG2.2.15 cell death. The reduction in intracellular ROS during treatment may be due to its antioxidant activity. Even though several reports have suggested that the generation of ROS can be considered beneficial in the treatment of cancer^46,47^, the ROS may stimulate a stem-like state of cancer cells, leading to a more aggressive form of cancer^48,49^. In this work, the reduced ROS during the cancer treatment might provide a new promising strategy for efficient cancer treatment. Assessment of two common death pathways including apoptosis and necrosis in Fe–TA NP treated HepG2.2.15 cells was also conducted using annexin V-FITC/PI staining assays to confirm the abovementioned observation regarding cell death mechanism. As expected, neither annexin V nor PI was stained in the HepG2.2.15 cells, suggesting that the cells did not die via apoptosis or necrosis (see Figure S7d). Compared to HepG2.2.15 cells, no large vesicular structures containing Fe–TA NPs or biologic materials were observed in the treated AML12 cells, but the key steps of autophagy and normal autophagic vacuoles such as autophagosome-lysosome fusion and autolysosome formation were found (Fig. 9b). In addition, it was found that Fe–TA NPs have no effect on overactivation of acidic organelles (Figure S8) and ROS (Figure S9). These results are consistent with the results of the toxicity test mentioned above.

**Figure 10:**
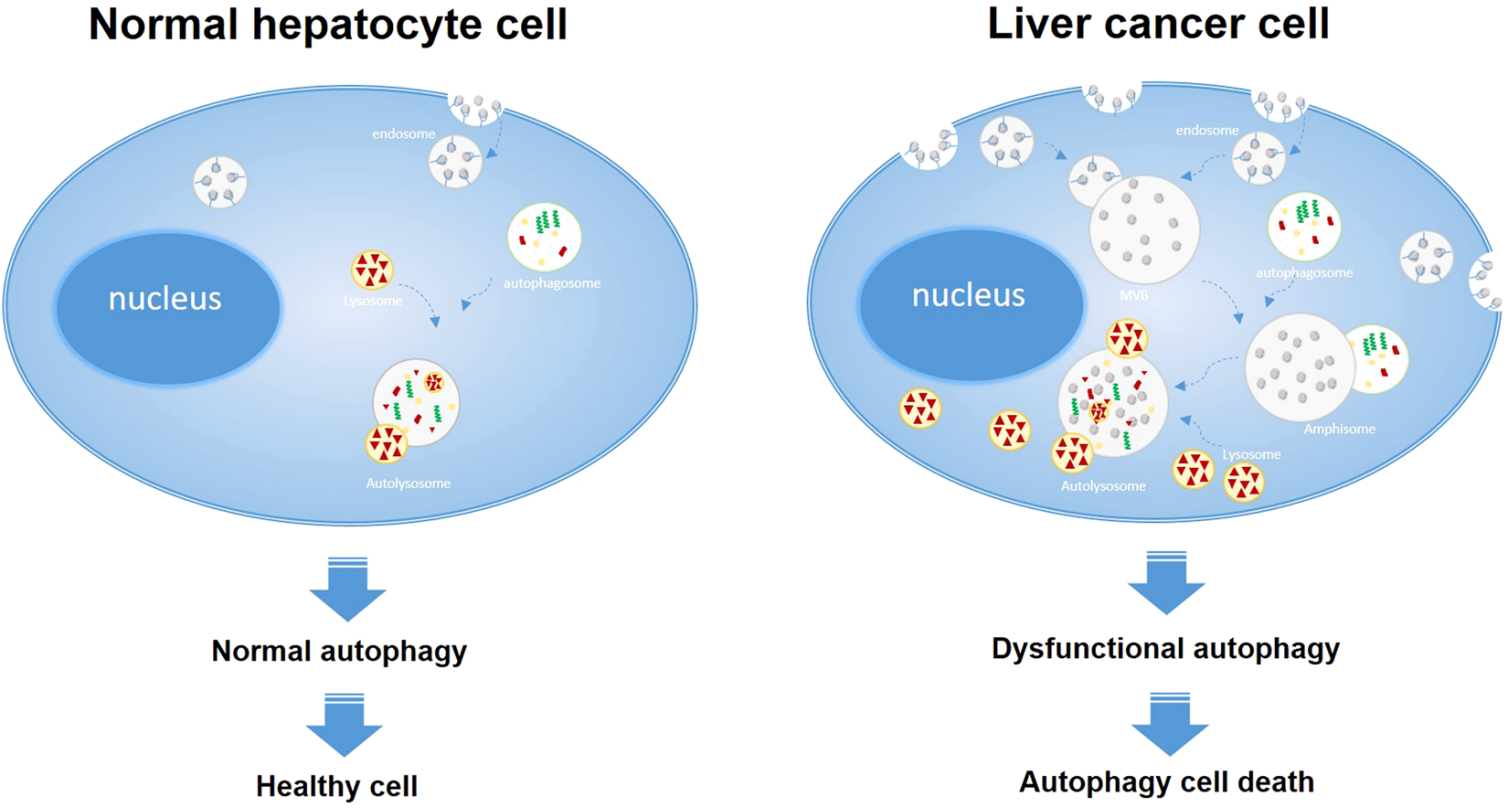
The proposed mechanism of cellular interaction and biological responses to Fe–TA NPs.

Based on the results obtained, possible mechanisms of Fe–TA NP induced autophagy in the two different cells are proposed (Fig. 10). In HepG2.2.15 cells, the much higher uptake of Fe–TA NPs by the cells via receptor-mediated endocytosis results in the formation of endosomal vesicles and thereafter multivesicular bodies (MVBs). Simultaneously, Fe–TA NPs, which might be regarded as autophagic cargos, trigger the autophagy process by initiating the formation of autophagosomes. Afterward, the MVBs are fused with mature autophagosomes to form amphisomes. The newly ingested materials in the amphisomes can be targeted for degradation by overregulation of the lysosomal function, implying excessive self-digestion which can result in autophagic cell death. This could be considered as one of the mechanisms of autophagic cell death induced by nanoparticles. However, the autophagy effect induced by Fe–TA NPs is manifested in a different way in the case of AML12 cells. Amphisomes, a combination of endosomes and autophagosomes, are hardly detected within the cells, which may be due to the much lower uptake efficiency. Major regulatory steps of normal autophagy were observed, including (i) autophagosome formation, (ii) autophagosome–lysosome fusion, and (iii) lysosomal degradation, indicating that Fe–TA NPs can be utilized as autophagy enhancers in normal hepatocytes.

### Fe–TA NPs are found to enhance imaging contrast of HepG2.2.15 cells in T_1_-weigthed MRI

**Figure 11:**
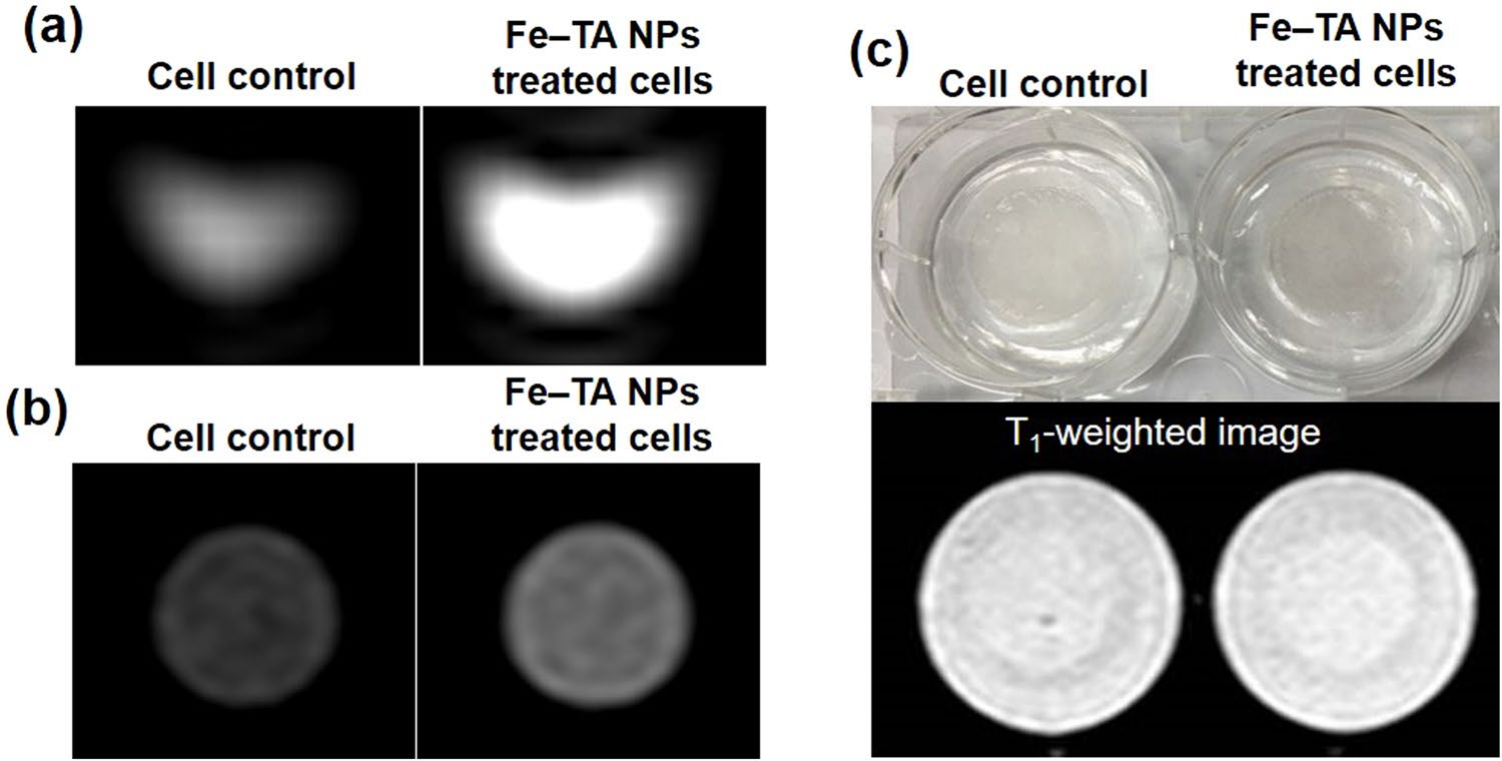
T_1_-weighted images of untreated and 100 µM Fe–TA NPs treated HepG2.2.15 cells treated for 24 h in three different imaging models: (**a**) cell pellets, (**b**) 4% acrylamide gel phantom, and (**c**) bio-scaffold.

Apart from the therapeutic potential of Fe–TA NPs, the possible use of Fe–TA NPs as imaging agents for enhancing the MRI signal was preliminarily investigated. Because of the high cellular uptake observed in HepG2.2.15 cells, cellular MR imaging experiments were conducted in three different imaging models including the cell pellet, acrylamide gel phantom, and polymeric bio-scaffold. In Fig. 11, as expected, it is clear that the T_1_-weighted images of the HepG2.2.15 cells treated with Fe–TA NPs in three different imaging models are brighter than those of the untreated cells. The enhancement in the signal intensity observed in the Fe–TA NP treated cells may be due to their higher accumulation within the cells. Moreover, the accumulation of the Fe–TA NPs in acidic compartments such as MVB, amphisomes, and autolysosomes is critical for enhancing the MRI signal because of the pH-dependent relaxivity of Fe–TA NPs^28^. Specifically, the dominated coordination states of the Fe–TA NPs are changed from the lower r_1_ state of Tris-dominated Fe–TA NPs to the higher r_1_ state of Bis-dominated Fe–TA NPs in such acidic compartments. Thus, they can be exploited as MR imaging agents for cancer cell imaging.

## Conclusion

This study has demonstrated that Fe–TA NPs are capable of inducing autophagy in different liver cell lines. The biological consequences of the inducing are depend on cellular uptake capability. A high uptake of Fe–TA NPs by HepG2.2.15 cells can result in autophagic cell death. Combined with the capability of enhancing the MRI signal, Fe–TA NPs offer a new promising approach for efficient imaging and treatment of liver cancer. Because of their good physicochemical properties along with autophagy-inducing properties, Fe–TA NPs have good potential for disease prevention and treatment mediated via modulation of the autophagy process. However, much effort is needed to confirm the possibility.

## Methods

### Materials and reagents

Ferric chloride hexahydrate (FeCl_3_.6H_2_O) was purchased from Fisher Chemicals and tannic acid (C_76_H_52_O_46_) was from Loba Chemie; Dulbecco’s modified Eagle’s medium (DMEM), Dulbecco’s modified Eagle’s medium Ham’s F-12 (DMEM/F-12), and Trypsin-EDTA were purchased from Caisson Laboratories. Fetal bovine serum (FBS) was from HyClone^TM^ Thermo Fisher Scientific. Collagen type I, ribonuclease A (Rnase A), triton X-100, propidium iodide (PI), and annexin V (FITC) apoptosis detection BioAssay™ kit were purchased from US Biological. Sodium azide (NaN_3_) and potassium thiocyanate (KSCN) were procured from BDH Laboratory. Dimethyl sulfoxide (DMSO) and monodensylcadavarine (MDC) were purchased from Sigma-Aldrich; nitric acid (HNO_3_) and ethanol were procured from ACL Labscan; and formalin was purchased from Gammaco. 3-dimethylthiazol-2,5-diphenyltetrazolium bromide (MTT) and acrylamide/bis-acrylaminde were purchased from Bio Basic Inc. and etramethylethylenediamine (TEMED) was procured from Invitrogen. Hydrochloric acid (HCl) was purchased from J.T. Baker; methanol was procured from Northern Chemicals and Glasswares Ltd.; and acridine orange was purchased from AMRESCO. LC3B polyclonal antibody and goat anti-rabbit lgG (H + L) secondary antibody, DyLight 488, were purchased from Thermo Fisher Scientific.

### Preparation of iron (III)-tannic acid nanoparticles (Fe–TA NPs)

The Fe–TA NPs were synthesized according to our previous report, with some modification^23^. In a typical experiment, the Fe–TA NPs were simply obtained by mixing ferric chloride and tannic acid in PBS buffer (pH 7.4) at room temperature for a few minutes in ambient air. The concentration of the Fe–TA NPs was expressed as equivalent concentration of iron, unless stated otherwise.

### Characterization of Fe–TA NPs

The UV-Vis absorbance of the samples including Fe^3+^, TA, and Fe–TA NPs was measured on a UV-Vis spectrophotometer (Agilent 8453) at wavelengths in the range of 400–800 nm. Functional groups and the Fe–O bonding were investigated by using a Fourier transform infrared (FTIR) spectrophotometer (BRUKER TENSOR27) and a Raman spectrophotometer (HORIBA JOBIN YVON, T64000). The morphology and the size were determined by using a transmission electron microscope (TEM, JEOL JEM-2100). The characteristic types of bonding found in the Fe–TA NPs were determined by X-ray photoelectron spectroscopy (Axis Ultra DLD, Kratos Analytical, Ltd.). The hydrodynamic diameter (HD) and the zeta potential (ZP) were measured by using a Malvern system (Malvern Instruments). The lipophilicity of the Fe–TA NPs was measured and reported as the partition coefficient (log P). The *r*_1_ MRI relaxivity of Fe–TA NPs in 4% acrylamide gel phantom was determined by using a Phillip Achiva 1.5 T MRI scanner at room temperature.

### Stability of Fe–TA NPs

In order to examine the transchelation and the transmetallation effects of the Fe–TA NPs, solutions of the Fe–TA NPs were challenged with various competitive ligands and metals. Typically, the Fe–TA NPs (0.2 µmol) were incubated with L-cysteine (1 µmol), 10% FBS solution, DMEM solution (without phenol red), Cu^2+^ (1 µmol), Zn^2+^ (1 µmol), and various oxyanions (1 µmol) for different lengths of incubation time. The transchelation and the transmetallation effects were monitored based on the decrease in the UV-Vis absorbance of the Fe–TA charge transfer band at 525 nm.

### Cell culture

The hepatitis B virus-infected hepatocellular carcinoma (HepG2.2.15 cells) and alpha mouse liver 12 (AML12 cells) cells were maintained in DMEM and DMEM/F12, supplemented with 10% FBS and 1% penicillin/streptomycin at 37 °C in a humidified atmosphere containing 5% CO_2_.

### Immunofluorescence staining of LC3B

HepG2.2.15 and AML12 cells were seeded in 6-well plates (3 × 10^5^ cells/well) and incubated overnight. After removing the culture media, the cells were further incubated with new culture media containing different amounts of samples including pure TA and Fe–TA NPs for 24 h. After washing, the cells were fixed in 4% formaldehyde for 15 min at room temperature and washed twice with PBS. Then, the cells were permeabilized in 100% cold methanol for 10 min at −20 °C. After washing, the cells were incubated with anti-LC3B antibody overnight at 4 °C. The following day, the cells were washed twice with PBS (5 min per wash) and further incubated with a secondary antibody solution consisting of a 1:500 dilution of Dylight 488 conjugated anti-rabbit secondary antibody for 2 h in the dark at room temperature. Finally, the cells were washed twice with PBS (5 min per wash) and analyzed under a fluorescence microscope (Olympus DP73, Japan).

### Cellular TEM analysis

HepG2.2.15 and AML12 cells were seeded in 6-well plates (1 × 10^6^ cells/well) and incubated overnight. The cells were then treated with Fe–TA NPs for 24 h. At the end of the treatment, the cells were washed twice with PBS, and pre-fixed with 2.5% glutaldehyde overnight. Then, post fixation with 1% osmium teroxide, dehydration with a series of ethanol solutions (70%, 80%, 90%, 95%, and 100%) and infiltration with propylene oxide/resin mixtures were consecutively performed. The cells were embedded in epoxy resin, which was polymerized at 60 °C for 2–3 days. Next, the sample blocks were thin-sectioned to a thickness in the range of 70–80 nm (Leica, Reichert Ultracut). The thin section was collected and stained with 2% uranyl acetate and lead citrate before examining under the TEM (JEM-2200FS) with an accelerating voltage of 200 kV.

### Cell viability assays

In this study, standard MTT and cell counting assays were used to evaluate the cytotoxicity of Fe–TA NPs. For the MTT assay, HepG2.2.15 and AML12 cells were seeded in 24-well plates (5 × 10^4^ cells/well) and incubated overnight. After that, the cells were treated with different concentrations of pure TA and Fe–TA NPs for 24 h. After washing, the cells were incubated with 5 mg/ml MTT solution in the incubator. After 4 h of incubation, the cells were washed twice with PBS, and the intracellular formazan was dissolved in DMSO. The absorbance of the formazan solution was measured by spectroscopy at 570 nm using DMSO as the blank. The cell viability (%) was expressed as the percentage relative to the control cells (untreated cells). For the cell counting assay, HepG2.2.15 and AML12 cells were seeded in 6-well plates (3 × 10^5^ cells/well) and incubated overnight. The cells were then treated with different concentrations of Fe–TA NPs for 24 h. After washing, the cells were detached by trypsinization and the number of viable cells was counted using a standard hemocytometer under an inverted light microscope (Nikon, TS2-FL).

### Cellular accumulation of Fe–TA NPs

HepG2.2.15 and AML12 cells were seeded in 6-well plates (3 × 10^5^ cells/well) and incubated overnight. The cells were then treated with different concentrations of Fe–TA NPs for different lengths of time. After washing, the cells were detached by trypsinization and centrifuged at 7000 rpm for 1 min. The resulting cell pellets were lysed with a mixed acid solution (1:1 of HCl:HNO_3_) for 30 min at 60 °C under sonication. After that, the lysed cells were made to spin down, and the supernatants were collected. The iron-containing supernatants were mixed with an excess amount of KSCN. Finally, the iron content was determined by measuring the absorbance of the iron-thiocyanate complex at 480 nm.

### Detection of acidic vesicular organelles with acridine orange (AO) and monodansylcadaverine (MDC)

In order to investigate the development of acidic organelles such as lysosomes, AO and MDC staining were performed. Although, in various reports, it has been mentioned that they were used to detect autophagosmes, they were demonstrated to have higher affinity for acidic vesicles such as lysosomes^50,51^. In a typical experiment, HepG2.2.15 and AML12 cells were seeded in 6-well plates (3 × 10^5^ cells/well) and incubated overnight. After that, the cells were treated with different concentrations of Fe–TA NPs for different lengths of time. After the treatment, the cells were washed twice with PBS and stained with appropriate concentrations of AO and MDC for 10 min. After washing, the red fluorescence of AO and the green fluorescence of MDC were analyzed by using a fluorescence microscope (Olympus DP73, Japan).

### Cell cycle distribution, reactive oxygen species (ROS), and annexin V-FITC/PI staining assays

To determine the cell cycle distribution, HepG2.2.15 cells were seeded in 6-well plates (3 × 10^5^ cells/well) and incubated overnight. The cells were treated with different concentrations of Fe–TA NPs for 24 h. The cells were washed twice with PBS and fixed in 70% ethanol for 24 h at 4 °C. The fixed cells were incubated with 5 μL of 10% Triton X, 50 μL of 2 mg/mL RNase A, and 5 μL of 1 mg/mL PI for 20 min at 37 °C. The DNA content was measured with a flow cytometer (Becton Dickinson, Switzerland) and the cell cycle distribution was analyzed by using Flowing Software 2.5.0.

For the ROS assay, HepG2.2.15 cells were seeded in 6-well plates (3 × 10^5^ cells/well) and incubated overnight. The cells were treated with 100 µM of Fe–TA NPs for different lengths of time. After the treatment, the cells were harvested by trypsinization and washed twice with PBS. The cells were then incubated with 0.1 µM H2DCFDA at 37 °C in an incubator for 30 min. The intracellular ROS was measured with the flow cytometer. The untreated cells were used as the control sample.

The annexin V-FITC/ PI staining assay was used to assess two common cell death pathways including apoptosis and necrosis. HepG2.2.15 cells were treated with 100 µM of Fe–TA NPs for 48 h. After washing, the cells were detached by trypsinization and further centrifuged at 7000 rpm for 1 min. Then, the cells were redispersed in a binding buffer and stained with FITC-conjugated annexin V and PI according to the manufacturer’s instructions. Finally, the cells were analyzed by using a flow cytometer.

### MRI cellular imaging

To investigate the feasibility of using Fe–TA NPs in the enhancement of the MRI signal in cancer cells, three different imaging models were set up, which included cell pellet, acrylamide gel phantom, and bio-scaffold models. In the cell pellet imaging model, HepG2.2.15 cells were seeded in 6-well plates (1 × 10^6^ cells/well) and incubated overnight. The cells were treated with 100 µM of Fe–TA NPs for 24 h. After the treatment, the cells were detached by trypsinization and centrifuged at 7000 rpm for 1 min. The cell pellets were washed twice with PBS and collected for imaging experiments. For the acrylamide gel phantom model, the treatment protocol was the same as that which was conducted in the cell pellet model but the treated HepG2.2.15 cells were redispersed in 4% acrylamide gel phantom for imaging. In the bio-scaffold model, the HepG2.2.15 cells (1 × 10^6^ cells) were grown on the scaffold for 30 days in the incubator. After washing, the cell-attached scaffold was incubated with 100 µM Fe–TA NPs for 24 h. After the treatment, the scaffold was washed twice with PBS and fixed with 4% formaldehyde for 15 min. The scaffold was placed on the top of 4% acrylamide gel previously cast in the 6-well plates. After that, the scaffold was covered with 4% acrylamide gel again. For the imaging experiment, T_1_-weighted imaging of three different cellular models was performed at room temperature using a Phillips Achieva 1.5 T MRI scanner. The imaging parameters were TR = 800 ms and TE = 10 ms.

## Electronic supplementary material

Supplementary information
