# Supplementary material for: Iron(III)-Tannic Molecular Nanoparticles Enhance Autophagy effect and T1 MRI Contrast in Liver Cell Lines
Source: Sci Rep. 2018 Apr 27;8:6647. doi: 10.1038/s41598-018-25108-1 (PMC5923259; doi:10.1038/s41598-018-25108-1)
Supplement: Supplementary file 1 — Supplementary information [file 41598_2018_25108_MOESM1_ESM.pdf]

Supplementary Information

Iron(III)-Tannic Molecular Nanoparticles Enhance Autophagy effect and T_1 MRI Contrast in Liver Cell Lines

Krungchanuchat Saowalak[1,2,+], Thongtem Titipun[2,*], Thongtem Somchai[3] , and Pilapong Chalermchai[1,*,+]

[1]Center of Excellence for Molecular Imaging (CEMI), Department of Radiologic Technology, Faculty of Associated Medical Sciences, Chiang Mai University, Chiang Mai 50200, Thailand
[2]Department of Chemistry, Faculty of Science, Chiang Mai University, Chiang Mai 50200, Thailand
[3]Department of Physics and Materials Science, Faculty of Science, Chiang Mai University, Chiang Mai 50200, Thailand
*chalermchai.pilapong@cmu.ac.th, ttpthongtem@yahoo.com
[+]these authors contributed equally to this work

Method

RT-PCR analysis. The HepG2.2.15 cells were treated with different concentrations of the Fe–TA NPs for different lengths of time. The total RNA was extracted using a NucleoSpin® RNA II (MACHEREY-NAGEL GmbH & Co KG). RNA concentration and purity was measured by ultraviolet spectrophotometer. A total of 0.8 µg RNA was used to synthesize cDNA using the RevertAidTM First Strand cDNA Synthesis kit (Thermo Fisher Scientific Inc.) according to the manufacturer's instructions. Quantitative PCR was performed with 2 µL of cDNA, 300 nM of each primer (Integrated DNA Technologies, Inc.) and 10 µL of SybrGreen qPCR Master Mix (SensiFAST^{TM} SYBR ® No-ROX One-step kits, BIOLINE) and analyzed with the LightCycler96 Software (Roche, Switzerland). The cycle threshold values were used to calculate the normalized expression of LC3 and β-actin using the LightCycler® 96 Software 1.1. The sequences of the primer pairs are listed below:
β-actin, 5'-TAG-TTGCGTTACACCCTTTCTTG-3'/ 5'-TCACCTTCA-CCGTTCCAGTT-3'
LC3, 5'-CATGA-GCGAGTTGGTCAAGAT-3'/ 5'-TCGTCTTTCTCCT-GCTCGTAG-3'

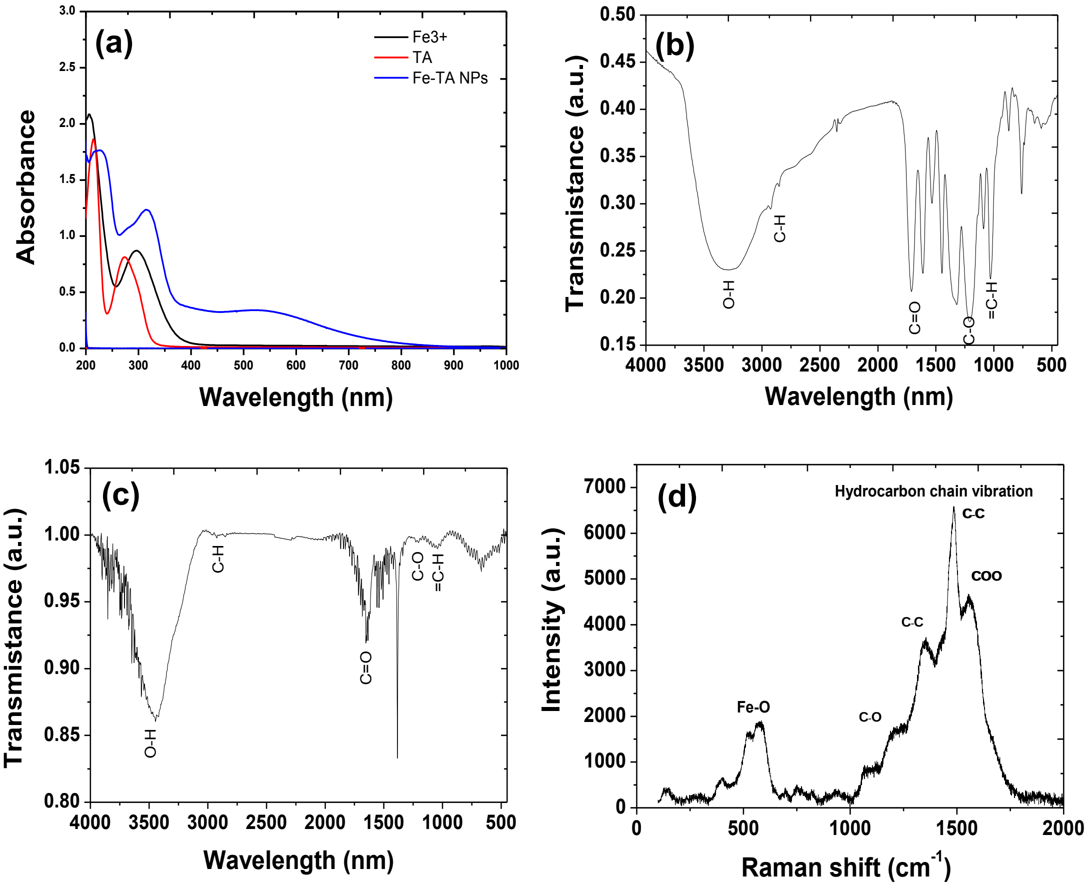

Figure S1. (a) UV-visible spectra of Fe(III), pure TA, and Fe–TA NPs; (b,c) FTIR spectra of pure TA acid and Fe–TA NPs; (d) Raman spectrum of Fe–TA NPs.

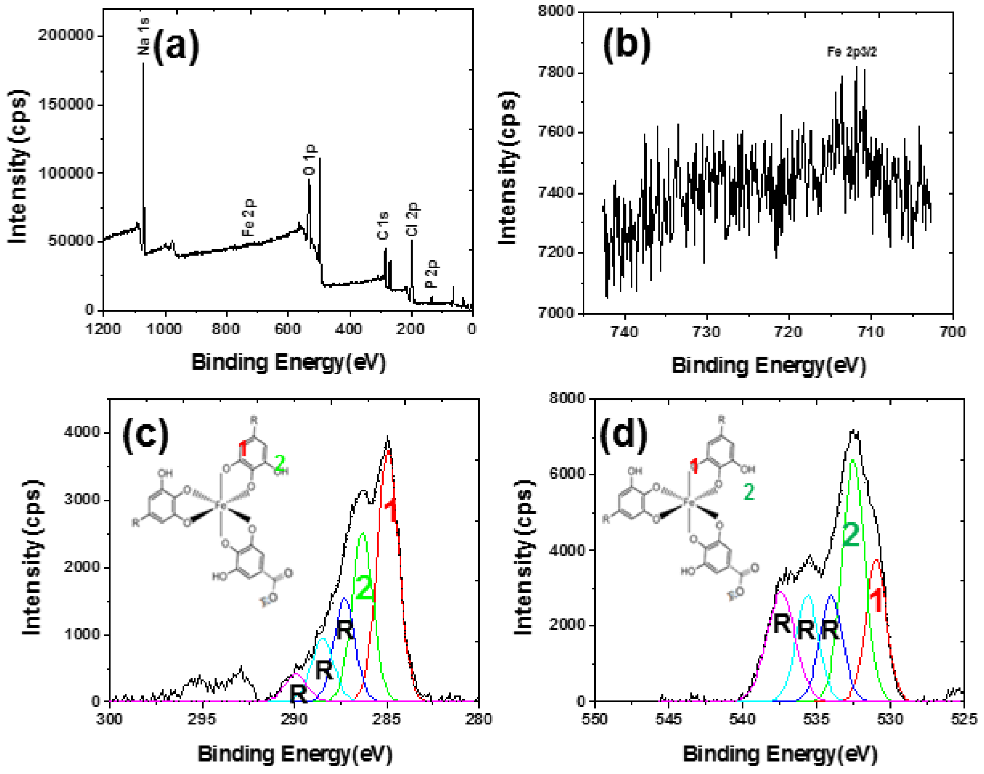

Figure S2. XPS analysis of the Fe–TA NPs. (a) Survey spectrum, (b) Fe 2p spectrum, (c) C 1s spectrum, and (d) O 1s spectrum.

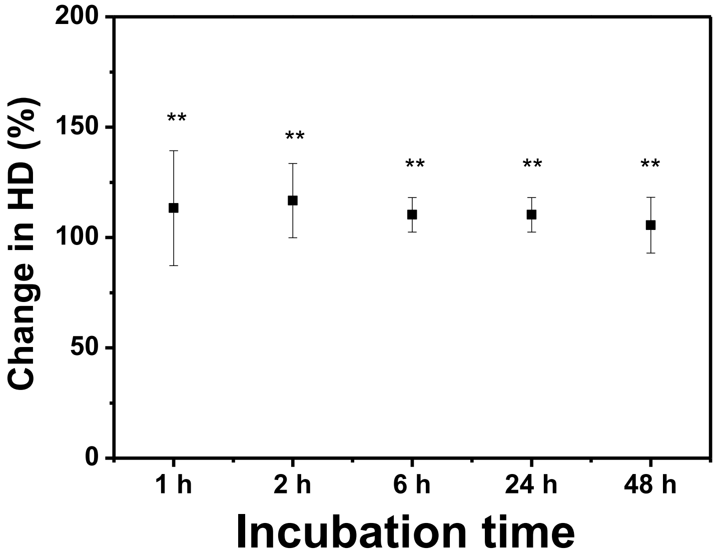

Figure S3. Time-dependent hydrodynamic size (HD) of the Fe–TA NPs in PBS containing 10% FBS buffer (** $p > 0.05$, ANOVA). (HDs of the Fe–TA NPs were normalized with respect to those measured in PBS (100%)).

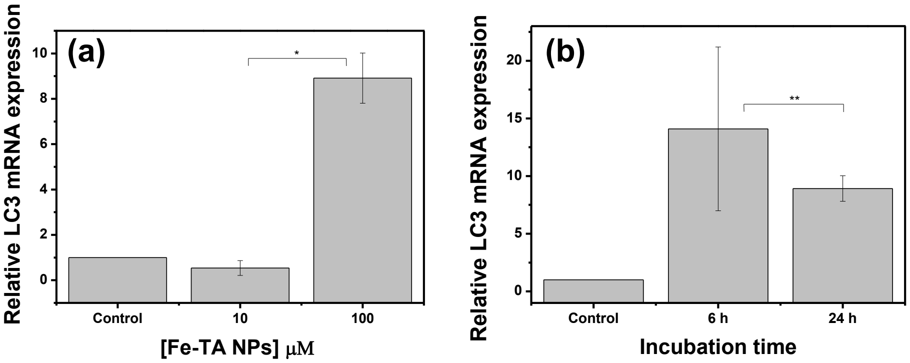

Figure S4. (a) The mRNA levels of LC3 in HepG2.2.15 cells treated with different concentrations of the Fe-TA NPs. (b) The mRNA levels of LC3 in HepG2.2.15 cells treated with 100 μM of the Fe-TA NPs for different lengths of time. (*p < 0.05, **p > 0.05).

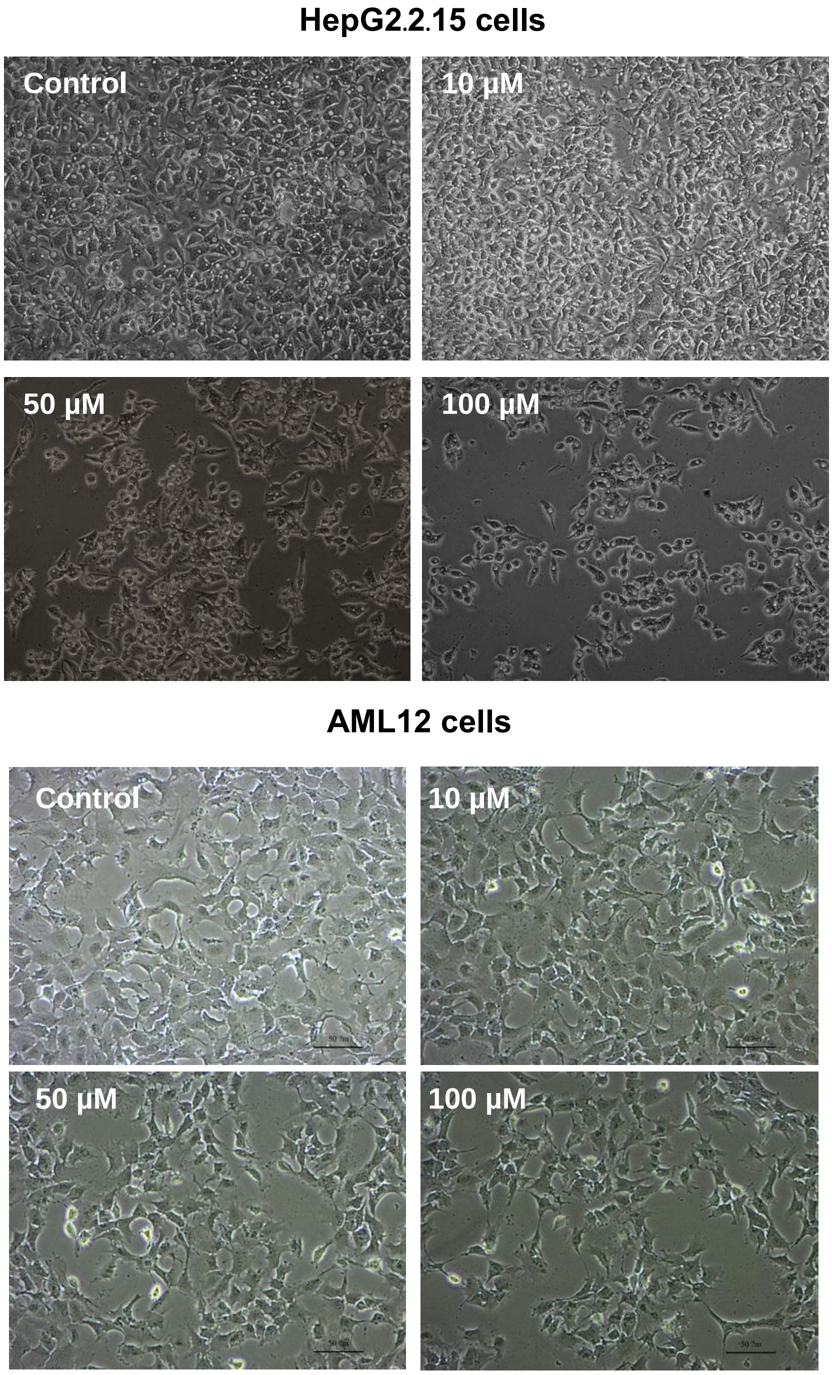

Figure S5. Cellular morphology analysis of (a) HepG2.2.15 cells and (b) AML12 cells after being treated with different concentrations of Fe–TA NPs for 24 h.

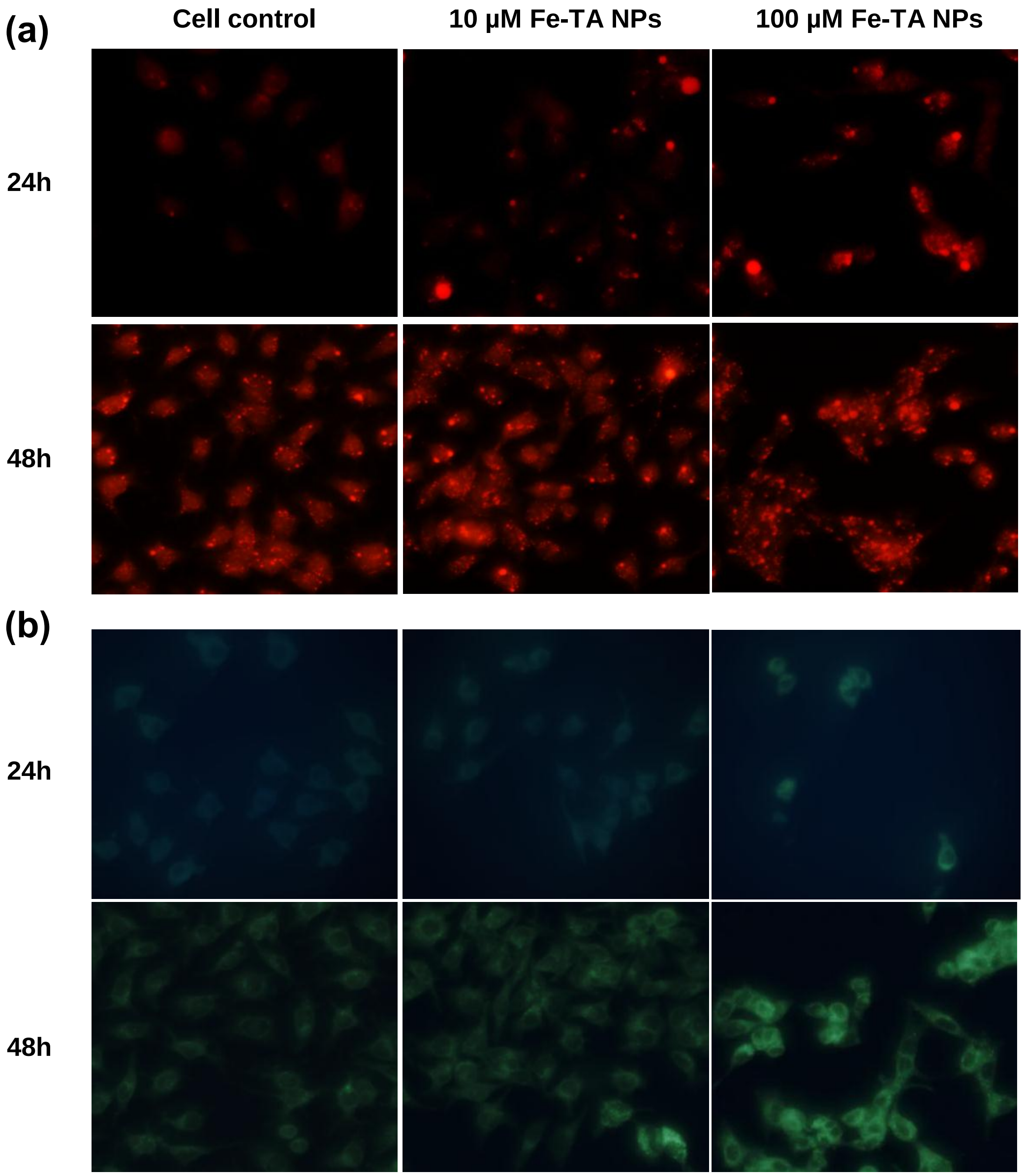

Figure S6. (a) Acridine orange (AO) staining and (b) monodansylcadaverine (MDC) staining of HepG2.2.15 cells after treatment with Fe–TA NPs for 24 h and 48 h.

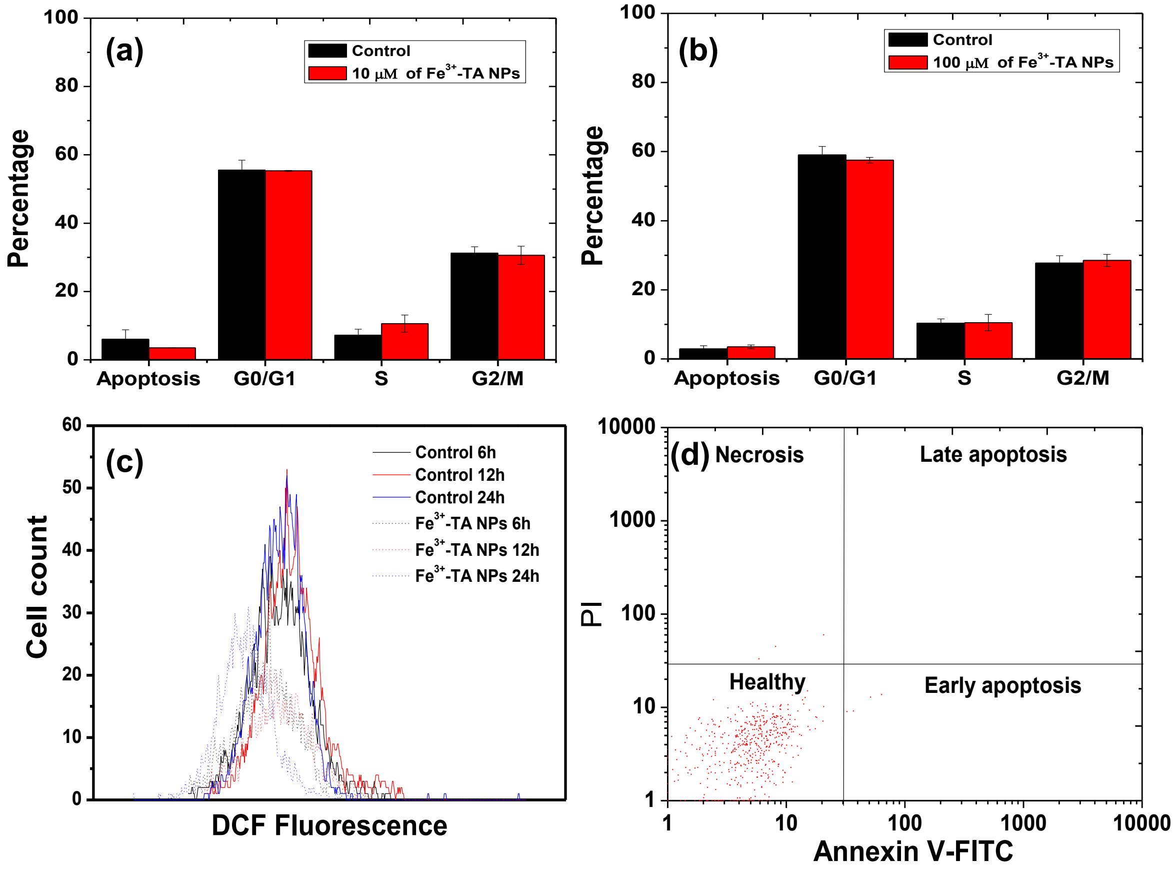

Figure S7. (a,b) Flow cytometric analysis of the cell cycle distribution, (c) flow cytometric analysis of the intracellular ROS, and (d) bi-parametric dot plot of annexin V-FITC/ propidium iodide (PI) co-staining (HepG2.2.15 cells).

.

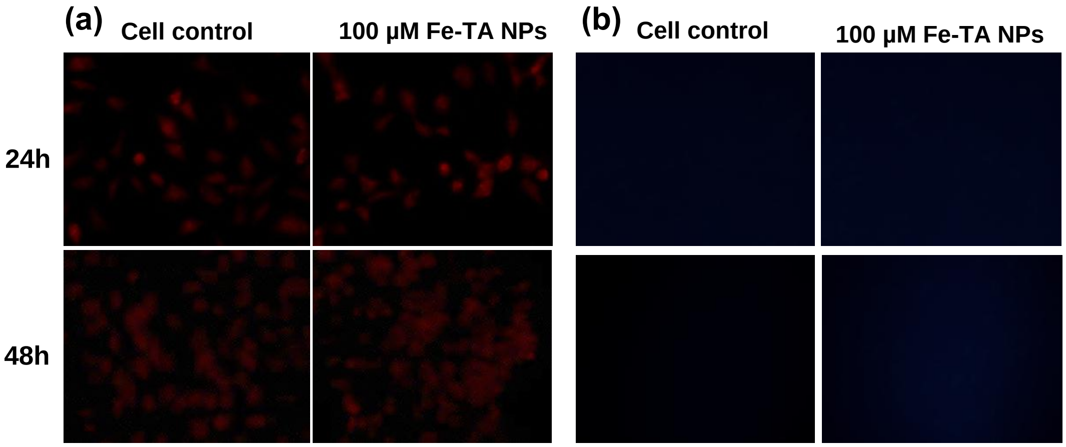

Figure S8. (a) Acridine orange (AO) staining and (b) monodansylcadaverine (MDC) staining of AML12 cells after treatment with Fe–TA NPs for 24 h and 48 h.

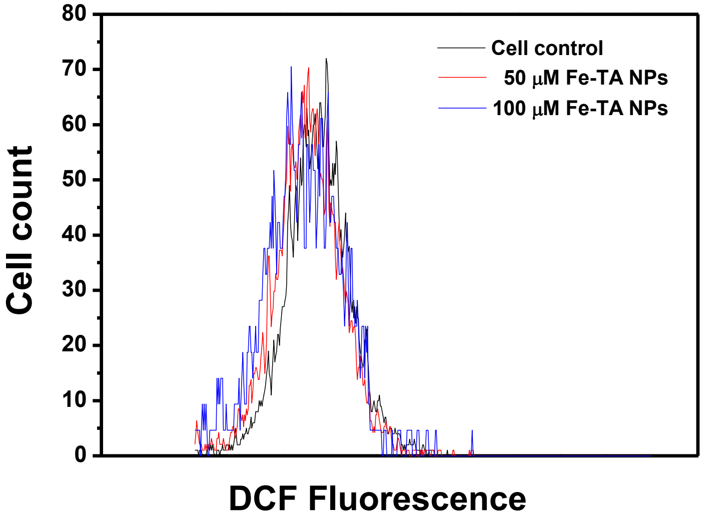

Figure S9. (c) flow cytometric analysis of the intracellular ROS of AML12 cells treated with different concentrations of Fe–TA NPs for 24 h.
